# Supplementary material for: Extraction and Purification of Flavonoids and Antiviral and Antioxidant Activities of Polygonum perfoliatum L
Source: Molecules. 2024 Dec 25;30(1):29. doi: 10.3390/molecules30010029 (PMC11721773; doi:10.3390/molecules30010029)
Supplement: Supplementary file 1 [file molecules-30-00029-s001.zip › molecules-3360828-supplementary.pdf]

Table S1: HPLC-MS/MS Analysis of 80 Flavonoid Components in Purified Flavonoids of *Polygonum perfoliatum* L

Molecular Weight (Da)	Formula	Ionization model	Compounds
302.04271	$C_{15}H_{10}O_7$	$[M+H]^+$ $[M-H]^-$	Quercetin
476.0959	$C_{22}H_{20}O_{12}$	$[M+H]^+$	Scutellarin methyl ester
344.09016	$C_{18}H_{16}O_7$	$[M+H]^+$ $[M-H]^-$	Lysionotin
610.15491	$C_{27}H_{30}O_{16}$	$[M+H]^+$ $[M-H]^-$	Rutin
578.16426	$C_{27}H_{30}O_{14}$	$[M+H]^+$ $[M-H]^-$	Rhoifolin
594.15981	$C_{27}H_{30}O_{15}$	$[M+H]^+$ $[M-H]^-$	Kaempferol-3-O-rutinoside
578.14318	$C_{30}H_{26}O_{12}$	$[M+H]^+$ $[M-H]^-$	Procyanidin B1
594.13799	$C_{30}H_{26}O_{13}$	$[M+H]^+$	Tiliroside
446.08528	$C_{21}H_{18}O_{11}$	$[M+H]^+$	Baicalin
344.08986	$C_{18}H_{16}O_7$	$[M+H]^+$ $[M-H]^-$	Eupatilin
290.07923	$C_{15}H_{14}O_6$	$[M+H]^+$ $[M-H]^-$	(+)-Catechin hydrate
478.07521	$C_{21}H_{18}O_{13}$	$[M+H]^+$ $[M-H]^-$	Quercetin 3-O-β-D-Glucuronide
594.15927	$C_{27}H_{30}O_{15}$	$[M+H]^+$ $[M-H]^-$	Vicenin II
594.15968	$C_{27}H_{30}O_{15}$	$[M+H]^+$ $[M-H]^-$	Lonicerin
320.05322	$C_{15}H_{12}O_8$	$[M+H]^+$ $[M-H]^-$	Dihydromyricetin
462.08013	$C_{21}H_{18}O_{12}$	$[M+H]^+$	Scutellarin
448.10109	$C_{21}H_{20}O_{11}$	$[M+H]^+$	Orientin
300.09991	$C_{17}H_{16}O_5$	$[M+H]^+$ $[M-H]^-$	4',7-Di-O-methylnaringenin

476.09606	$C_{22}H_{20}O_{12}$	$[M+H]^+$	6-O-Methylscutellarin
432.1061	$C_{21}H_{20}O_{10}$	$[M+H]^+$ $[M-H]^-$	Vitexin
594.15942	$C_{27}H_{30}O_{15}$	$[M+H]^+$	Kaempferol 3-glucorhamnoside
448.10126	$C_{21}H_{20}O_{11}$	$[M+H]^+$ $[M-H]^-$	Homoorientin
402.1316	$C_{21}H_{22}O_8$	$[M+H]^+$	Nobiletin
448.10091	$C_{21}H_{20}O_{11}$	$[M+H]^+$ $[M-H]^-$	Astragalin
302.04281	$C_{15}H_{10}O_7$	$[M+H]^+$ $[M-H]^-$	Morin
304.0584	$C_{15}H_{12}O_7$	$[M+H]^+$ $[M-H]^-$	Taxifolin
316.05834	$C_{16}H_{12}O_7$	$[M+H]^+$ $[M-H]^-$	Isorhamnetin
592.18118	$C_{28}H_{32}O_{14}$	$[M+H]^+$	Linarin
272.06851	$C_{15}H_{12}O_5$	$[M+H]^+$ $[M-H]^-$	Naringenin
286.08443	$C_{16}H_{14}O_5$	$[M+H]^+$ $[M-H]^-$	Isosakuranetin
464.09548	$C_{21}H_{20}O_{12}$	$[M+H]^+$	Hyperoside
464.09548	$C_{21}H_{20}O_{12}$	$[M-H]^-$	Isoquercitrin
432.10604	$C_{21}H_{20}O_{10}$	$[M+H]^+$	Afzelin
290.07915	$C_{15}H_{14}O_6$	$[M+H]^+$ $[M-H]^-$	Epicatechin
286.0478	$C_{15}H_{10}O_6$	$[M+H]^+$	Luteolin
316.05843	$C_{16}H_{12}O_7$	$[M+H]^+$ $[M-H]^-$	Eupafolin
564.14855	$C_{26}H_{28}O_{14}$	$[M+H]^+$	Isoschaftoside
314.07915	$C_{17}H_{14}O_6$	$[M+H]^+$ $[M-H]^-$	Pectolinarigenin
286.04786	$C_{15}H_{10}O_6$	$[M+H]^+$ $[M-H]^-$	Kaempferol
608.1755	$C_{28}H_{32}O_{15}$	$[M+H]^+$	Diosmin

302.07947	$C_{16}H_{14}O_6$	$[M+H]^+$ $[M-H]^-$	Hesperetin
318.03759	$C_{15}H_{10}O_8$	$[M+H]^+$ $[M-H]^-$	Myricetin
506.10665	$C_{23}H_{22}O_{13}$	$[M+H]^+$	6''-O-Acetylisoquercitrin
288.06346	$C_{15}H_{12}O_6$	$[M+H]^+$ $[M-H]^-$	Eriodictyol
434.08531	$C_{20}H_{18}O_{11}$	$[M+H]^+$	Avicularin
434.1217	$C_{21}H_{22}O_{10}$	$[M+H]^+$ $[M-H]^-$	Engeletin
374.10122	$C_{19}H_{18}O_8$	$[M+H]^+$ $[M-H]^-$	Chrysosplenetin B
298.08442	$C_{17}H_{14}O_5$	$[M+H]^+$	5-Hydroxy-6,7-dimethoxy lflavone
318.07417	$C_{16}H_{14}O_7$	$[M+H]^+$	Padmatin
482.12195	$C_{25}H_{22}O_{10}$	$[M+H]^+$ $[M-H]^-$	Silibinin
610.15338	$C_{27}H_{30}O_{16}$	$[M+H]^+$ $[M-H]^-$	2''-O-β-L-Galactopyranosy lorientin
520.08531	$C_{23}H_{20}O_{14}$	$[M+H]^+$ $[M-H]^-$	2-Hydroxy-4-(9-hydroxy-7-methoxy-8-oxo-8H-[1,3]dioxolo[4,5-g]chromen-6-yl)phenyl β-D-glucopyranosiduronic acid
270.08921	$C_{16}H_{14}O_4$	$[M+H]^+$	Medicarpin
416.11073	$C_{21}H_{20}O_9$	$[M+H]^+$ $[M-H]^-$	Puerarin
284.06847	$C_{16}H_{12}O_5$	$[M+H]^+$ $[M-H]^-$	Glycitein
270.05282	$C_{15}H_{10}O_5$	$[M+H]^+$ $[M-H]^-$	Genistein
268.03717	$C_{15}H_8O_5$	$[M+H]^+$	Coumestrol
430.12638	$C_{22}H_{22}O_9$	$[M+H]^+$	Ononin
330.07395	$C_{17}H_{14}O_7$	$[M+H]^+$	Iristectorigenin B

600.1645	$C_{34}H_{24}N_4O_7$	$[M+H]^+$	6-[Bis(5-nitro-1H-indol-3-yl)methyl]-3-(4-methoxyphenyl)-8-methyl-2H-chromen-2-one
358.10525	$C_{19}H_{18}O_7$	$[M-H]^-$	Retusin (flavonol)
286.08412	$C_{16}H_{14}O_5$	$[M-H]^-$	5,7-Dihydroxy-4-(4-methoxyphenyl)-2-chromanone
450.11621	$C_{21}H_{22}O_{11}$	$[M-H]^-$	3,5-Dihydroxy-2-(4-hydroxyphenyl)-4-oxo-3,4-dihydro-2H-chromen-7-yl hexopyranoside
360.08452	$C_{18}H_{16}O8$	$[M-H]^-$	5,7,3'-Trihydroxy-6,4',5'-trimethoxyflavone
442.09	$C_{22}H_{18}O_{10}$	$[M-H]^-$	(-)-Epicatechin gallate
290.07904	$C_{15}H_{14}O_6$	$[M-H]^-$	Cianidanol
436.13695	$C_{21}H_{24}O_{10}$	$[M-H]^-$	Phloridzin
270.05282	$C_{15}H_{10}O_5$	$[M-H]^-$	Apigenin
446.08491	$C_{21}H_{18}O_{11}$	$[M-H]^-$	Apigenin 7-O-glucuronide
300.027	$C_{15}H_8O_7$	$[M-H]^-$	Demethylwedelolactone
448.10056	$C_{21}H_{20}O_{11}$	$[M-H]^-$	Kaempferol-7-O-β-D-glucopyranoside
462.11621	$C_{22}H_{22}O_{11}$	$[M-H]^-$	Tectoridin
256.07356	$C_{15}H_{12}O_4$	$[M-H]^-$	Pinocembrin
564.14791	$C_{26}H_{28}O_{14}$	$[M-H]^-$	Vicenin III
300.06339	$C_{16}H_{12}O_6$	$[M-H]^-$	Diosmetin
284.06847	$C_{16}H_{12}O_5$	$[M-H]^-$	Genkwanin
448.13695	$C_{22}H_{24}O_{10}$	$[M-H]^-$	Isosakuranin
434.1213	$C_{21}H_{22}O_{10}$	$[M-H]^-$	Prunin

578.14243	$C_{30}H_{26}O_{12}$	$[M-H]^-$	Procyanidin B2
330.07395	$C_{17}H_{14}O_7$	$[M-H]^-$	Jaceosidin
